# Cell Cycle-Dependent Turnover of 5-Hydroxymethyl Cytosine in Mouse Embryonic Stem Cells

**DOI:** 10.1371/journal.pone.0082961

**Published:** 2013-12-10

**Authors:** Junji Otani, Hironobu Kimura, Jafar Sharif, Takaho A. Endo, Yuichi Mishima, Toru Kawakami, Haruhiko Koseki, Masahiro Shirakawa, Isao Suetake, Shoji Tajima

**Affiliations:** 1 Graduate School of Engineering, Kyoto University, Katsura, Nishikyo-ku, Kyoto, Japan; 2 Institute for Protein Research, Osaka University, Suita, Osaka, Japan; 3 RIKEN Center for Integrative Medical Sciences, Tsurumi-ku, Yokohama, Kanagawa, Japan; 4 CREST, Japan Science and Technology Agency, Kawaguchi, Saitama, Japan; Chinese Academy of Science, China

## Abstract

Hydroxymethylcytosine in the genome is reported to be an intermediate of demethylation. In the present study, we demonstrated that maintenance methyltransferase Dnmt1 scarcely catalyzed hemi-hydroxymethylated DNA and that the hemi-hydroxymethylated DNA was not selectively recognized by the SRA domain of Uhrf1, indicating that hydroxymethylcytosine is diluted in a replication-dependent manner. A high level of 5-hydroxymethylcytosine in mouse embryonic stem cells was produced from the methylcytosine supplied mainly by *de novo*-type DNA methyltransferases Dnmt3a and Dnmt3b. The promoter regions of the *HoxA* gene cluster showed a high hydroxymethylation level whilst the methylcytosine level was quite low, suggesting that methylated CpG is actively hydroxylated during proliferation. All the results indicate that removal and production of hydroxymethylcytosine are regulated in replication-dependent manners in mouse embryonic stem cells.

## Introduction

Methylation of cytosine in CpG sequences is an important epigenetic modification for the regulation of gene expression. Global DNA methylation patterns are established by *de novo*-type DNA methyltransferases Dnmt3a and Dnmt3b at an early stage of embryogenesis [1]. Dnmt3a and Dnmt3b partly compensate for each other during embryogenesis as the phenotype is more severe in double knockout embryos [1]. Different from Dnmt1, these two enzymes show no preferential DNA methylation activity towards hemi-methylated DNA [2,3]. Mouse embryonic stem cells (mESCs), which mimic embryonic proper cells at a stage around implantation, highly express Dnmt3a2, which lacks the N-terminal 219 amino acid residues in mice, and Dnmt3b compared to differentiated somatic cells [4,5]. Once the DNA methylation patterns are established, they are faithfully propagated to the next generation by maintenance-type DNA methyltransferase Dnmt1 in a cell lineage-dependent manner [6]. Although Dnmt1 shows maintenance methylation activity by itself *in vitro* [7], another factor, Uhrf1 (Np95), is necessary for the maintenance methylation in mESCs [8]. The SRA (SET and Ring finger Associated) domain in Uhrf1 specifically binds hemi-methylated DNA and flips the methylated cytosine out of the double-stranded DNA [9-11]. 

On the contrary, the players in DNA demethylation have not been completely elucidated yet [12,13]. Recently, hydroxymethylcytosine (5hmC) produced from methylcytosine (5mC) through DNA dioxygenase ten-eleven translocation (Tet) was found to be an intermediate of demethylation [14,15]. Genome-wide analyses demonstrated that 5hmC is abundant at promoters and transcription start sites (TSS) [16-20], suggesting that 5hmC can be a sign of transcriptional regulation. The 5hmC enrichment shows correlation with the bivalent modifications on K4 and K27 methylation of histone H3 [17,21]. In agreement with this, many of the Tet1, one of the three isoforms of Tet, target genes are occupied by polycomb repressive complex 2 (PRC2) [18,21]. Tet1 and Tet2 are highly expressed in mESCs, and are rapidly down regulated upon differentiation, while Tet3 is highly expressed in oocytes and zygotes [22,23]. 

In the present study, we demonstrated that Dnmt1 scarcely methylated hemi-hydroxymethylated DNA and that the SRA domain of Uhrf1 could not specifically bind hemi-hydroxymethylated DNA. As a result, 5hmC is diluted after replication, indicating that global demethylation occurs passively in mESCs. A high level of 5hmC in mESCs is maintained through the cooperation of *de novo*-type DNA methyltransferase, Dnmt3a (Dnmt3a2) and Dnmt3b, and Tet dioxygenase. Turnover of 5hmC in mESCs is regulated in a cell cycle-dependent manner. 

## Materials and Methods

### Cell culture

All the mESCs, i.e. parent J1 [6], *Dnmt1* [24], *Dnmt3a* [1], *Dnmt3b* [1], *Dnmt3a* and *Dnmt3b* double [1], and *Dnmt1*, *Dnmt3a*, and *Dnmt3b* triple knockout [25] ones, were kindly provided by Dr. Masaki Okano (CDB, RIKEN, Kobe). J1 and mutant mESCs were cultured in Glasgow minimum essential medium supplemented with sodium pyruvate, non-essential amino acids, 0.1 mM 2-mercaptoethanol, leukemia inhibitory factor, and 15% (v/v) Knockout-Serum Replacement (Invitrogen). 

### Isolation of cells at different stages of the cell cycle

Three different techniques, involving inhibitors, FACS, and cell cycle synchronization, respectively, were employed for the enrichment of cells at different stages of the cell cycle. To arrest cells at the S-phase, they were treated with 5 μM aphidicolin or 1 mM hydroxyurea. To arrest cells at the G1/G0-phase, 15% (v/v) KSR was replaced by 1% (v/v) fetal bovine serum (Intergen) as described elsewhere [26]. To arrest cells at the G2/M-phase, 200 ng/ml of nocodazole (Sigma) was added to the medium, followed by culturing for 24 h before determination of 5hmC.

 For sorting the cells by FACS, mESCs were EDTA- and trypsin-treated, and suspended in Dulbecco’s phosphate-buffered saline (PBS). The cells were fixed with 70% ethanol, and then stained with propidium iodide [27] and sorted with a BD FACSAria (Becton and Dickinson). Genomic DNA was isolated from the cells at the G1, S, and G2/M phases, and then the 5hmC contents were determined.

The mESCs were synchronized by the double thymidine block method [27]. In brief, mESCs were treated with 2.5 mM thymidine for 12 h and washed twice with PBS, and then the culture was continued in the normal medium for 9 h. After that, the cells were treated again with 2.5 mM thymidine for 14 h. After washing the cells with PBS, the culture was restarted in a normal medium. The cells were collected at the indicated times, and then the DNA content in the cells was determined by FACS, followed by determination of the 5hmC content.

### DNA methylation activity

 Recombinant mouse Dnmt1, Dnmt3a, and Dnmt3b were prepared and determined the methylation activity as described elsewhere [3,28]. In brief, 13.2, 39.3, or 41.1 nM recombinant Dnmt1, Dnmt3a, or Dnmt3b, respectively, was incubated with 25 nM synthesized unmethylated, hemi-methylated, or hemi-hydroxymethylated 35-bp DNA, and 2.2 μM [^3^H]-*S*-adenosyl-L-methionine (SAM) (10 Ci/mmol; Perkin Elmer) in 25 μl of buffer comprising 2.7 M glycerol, 5 mM EDTA, 0.2 mM DTT, 25 mM NaCl, and 20 mM Tris–HCl, pH 7.4, at 37°C for the indicated times. The synthesized DNAs, 5’-GGCAATCAGTTCACTTCGAGCCCAGGTATTTAGCC-3’ and 5’-GGCTAAATACCTGGGCTXGAAGTGAACTGATTGCC-3’, where X was C, 5mC, or 5hmC, were annealed and served for DNA methylation activity measurements. The radioactivity incorporated into DNA was determined with a scintillation counter, and the amount of methyl-group transferred to the DNA was calculated from the specific activity of [^3^H]-SAM. 

### RT-PCR

Using total RNA prepared with TRIzol (Invitrogen), a cDNA library was prepared with Superscript II reverse transcriptase (Invitrogen) and random hexamers. The optimized PCR conditions, and the primer sets for Tet1, Tet2, Tet3, and Gapdh are shown in Table S1. The amplified products were resolved on 2% agarose gels and visualized by ethidium bromide staining.

### DNA-binding assay

 The recombinant SRA domain, 405-613, of mouse Uhrf1 was prepared as described elsewhere [9]. The oligonucleotides used for the binding assay were of 12-bp in length with 5’-CTACCGGATTGC-3’ and 5’-GCAATCXGGTAG-3’, where X was C, 5mC, or 5hmC. These 12-bp oligonucleotides were annealed to form unmethylated (CG/CG), hemi-methylated (CG/5mCG), and hemi-hydroxymethylated (CG/5hmCG) duplexes. To prepare ^32^P-labeled DNA for the competition experiments, the first strand was 5’-end labeled with T4 polynucleotide kinase (Toyobo) and [γ-^32^P]-ATP (Muromachi Kagaku, Tokyo) before the annealing. 

 For the gel mobility shift assays, 0-3 μM recombinant SRA was incubated with 1 μM DNA in the presence of 250 ng of poly (dI-dC)(dI-dC) duplex (Sigma) at 4°C for 30 min in a buffer comprising 0.1 M NaCl, 0.1 mM TCEP, and 25 mM HEPES-NaOH, pH 7.4. After the incubation, the mixtures were electrophoresed in 7.5% native polyacrylamide gels in 1x TBE, stained with GelGreen (Biotium, Inc.), and then visualized with a BAS 7000 (Fuji Film). For the competition assays, 5 μM recombinant SRA and 1 μM ^32^P-labeled CG/5mCG were incubated with 0-10 μM un-labeled CG/CG, CG/5mCG, or CG/5hmCG in the presence of 250 ng poly (dI-dC)(dI-dC) duplex at 4°C for 30 min. The samples were electrophoresed as above and the radio-labeled bands were visualized with a BAS2000 phosphor imager (Fuji Film).

### Quantification of 5hmC and 5mC

The determination of 5hmC was performed as described elsewhere [29] with slight modifications. The cDNA of β-glucosyltransferase (β-GT) used in the procedure was isolated by PCR using T4 phage genomic DNA as the template. The cDNA of β-GT was subcloned into pET28, expressed in BL21-CodonPlus(DE3)-RIL *Escherichia coli*, and purified with Ni-NTA Sepharose. In brief, 200 ng of genomic DNA was incubated with 0.4 μM β-GT and 33.4 μM [^3^H]-UDP-glucose (60 Ci/mmol, Perkin Elmer) at 25°C for 1 h in 25 μl of reaction buffer comprising 50 mM potassium acetate, 10 mM magnesium acetate, 1 mM DTT, and 20 mM Tris-acetate, pH 7.9. At the end of the incubation, 20 μg of Proteinase K was added to the mixture, followed by incubation in 1% (w/v) SDS at 55°C for 30 minutes. After the incubation, the reaction mixture was spotted onto a DE81 filter disc (GE Healthcare). The disc was washed as described elsewhere [3], and radioactivity incorporated into DNA was determined with a scintillation counter. The relative hydroxymethylation levels were calculated from the standard curve of 200 ng of non-hydroxymethylated DNA with 0-1 ng of hydroxymethylated DNA added (Figure S1A). Unmodified or hydroxymethylated DNA was prepared by PCR, using the histone H3 gene in pBlueScript as the template in the presence of dCTP or deoxy-hydroxymethyl CTP (5hmCTP) with the specific primer set complementary to the multi-cloning site of pBlueScript, respectively.

For determination of the 5mC content, 200 ng of genomic DNA was incubated with 2 units of M.SssI (Fermentas, Thermo Fisher Scientific) and 2.8 μM [^3^H]-SAM (10 Ci/ mmol; Perkin Elmer) at 37°C in 20 μl of reaction buffer comprising 50 mM NaCl, 10 mM MgCl_2_, 1 mM dithiothreitol, 10 mM Tris-HCl, pH 7.9. After 1 h incubation, the radioactivity incorporated into the genomic DNA was determined with a scintillation counter. The relative methylated DNA contents were calculated from the standard curve (Figure S1B). Fully methylated DNA was prepared by M.SssI treatment of the unmodified DNA as above. The methylation efficiency with M.SssI was more than 95%.

### Enrichment of 5mC- or 5hmC-containing DNA

Cells (1x 10^7^) were treated with 100 μg Proteinase K in a buffer comprising 0.5% SDS, 0.1 M EDTA, and 10 mM Tris-HCl, pH 8.0, at 50°C overnight. Genomic DNA was purified by phenol/chloroform extraction and precipitation with ethanol as described elsewhere [30]. Purified DNA was dissolved in 1x TE, and then fragmented into 200-1000 bp fragments by sonication (on 15 sec, off 15 sec, and total 20 min) with a Bioruptor (Cosmobio, Tokyo). 

Selective precipitation of the DNA fragments containing 5hmC was performed as described elsewhere [31]. In brief, 10 μg of sonicated DNA was treated with 0.2 μM β-GT and 250 μM UDP-6-N_3_-glucose at 37°C for 1 h. Glucosylated DNA was reacted with 150 μM dibenzocyclooctyne-modified biotin by click chemistry. Biotinylated DNA was captured with Dynabeads M-280 streptavidin (Invitrogen). The hydroxylated histone H3, which was prepared as described under “Quantification of 5hmC and 5mC”, was biotinylated by click chemistry. The efficiency of pull-down of the biotinylated DNA (2 pg) from the mixture with genome DNA (10 μg) was 43%, as determined by q-PCR.

Precipitation of the DNA fragments containing 5mC was performed as described previously [32] with a slight modification. In brief, 10 μg of sonicated DNA was incubated with 1.2 μg of recombinant His-GST-MBD1 coding 1-75 of MBD1 [33] and MagneGST beads (Promega) at 4°C overnight. Bound DNA was eluted by proteinase K treatment at 50°C for 3 h. The eluted DNA was further purified by phenol-chloroform extraction followed by ethanol precipitation, and then dissolved in 1x TE. The efficiency of pull-down of the methylated histone H3 DNA fragments (2 pg) prepared as described under “Quantification of 5hmC and 5mC” from the mixture with genome DNA (10 μg) was 90%, as determined by q-PCR.

The DNA fragments enriched with 5mC or 5hmC DNA were hybridized with mouse 2x 105 k CpG island microarrays (Agilent, #G4811A). The DNA fragments of 500 ng or after amplification by *in vitro* transcription using 50 ng as the starting material as described elsewhere [34] were labeled with either Cy3 or Cy5. The labeled materials were hybridized according to the supplier’s protocol. The log ratios of the signals of input fragments (Cy3-labeled) and the fragments after precipitation enriched with 5mC or 5hmC (Cy5-labeled) were analyzed for murine CpG islands. The Gene Expression Omnibus accession number for the 5mC and 5hmC reported in this paper is GSE51473.

The specific genome regions enriched with 5mC and 5hmC were quantified by qPCR with Thunderbird SYBR qPCR Mix (Toyobo). A list of the primer sets used for qPCR is presents as Table S2. For *HoxA7* and *Oct4*, the primer sequences were taken from elsewhere [35].

### Chromatin immunoprecipitation (ChIP) and qPCR

ChIP was performed as described previously [36] with slight modifications. In brief, cells were fixed in 1.0% or 1.5% formaldehyde for the precipitation of Tet and Dnmt1 or Dnmt3a and Dnmt3b, respectively, for 10 min at room temperature, which was terminated with 125 mM glycine. The DNA was fragmented into 200-1,000 bp fragments by sonication. Solubilized chromatin was incubated with mouse monoclonal IgG (Cat No. 12-371, Millipore), rabbit IgG (Cat No. 12-370, Millipore), anti-Dnmt1 mouse monoclonal Dnmt1 (clone: 60B1220.1, Cat No. IMG-261A, Imgenex), anti-Dnmt3a/3a2 [5], anti-Dnmt3b [2], anti-Tet1 (Cat. No. 09-872, Millipore), anti-Tet2 (Cat No. R1086-6b, Abiocode), or anti-Tet3 (Cat. No. 61395, Activemotif) antibodies at 4°C overnight. The DNA-protein complexes were purified with Dynabeads Protein G (Invitrogen) or Dynabeads anti-mouse IgG (Invitrogen). Cross-linking was reversed by overnight mixing in a Thermomixer (Thermo) at 65°C, and then the DNA was treated with RNaseA and Proteinase K. DNA was purified by phenol-chloroform extraction and ethanol precipitation, and then dissolved in 1x TE. Enrichment of the immuno-precipitated Dnmt1, Dnmt3a/3a2, Dnmt3b, Tet1, Tet2, and Tet3 was quantitated by qPCR with Thunderbird SYBR qPCR Mix. A list of the primer sets is presented as Table S2. 

## Results

### 5-Hydroxymethylcytosine is efficiently diluted during replication in mESCs

**Figure 1:**
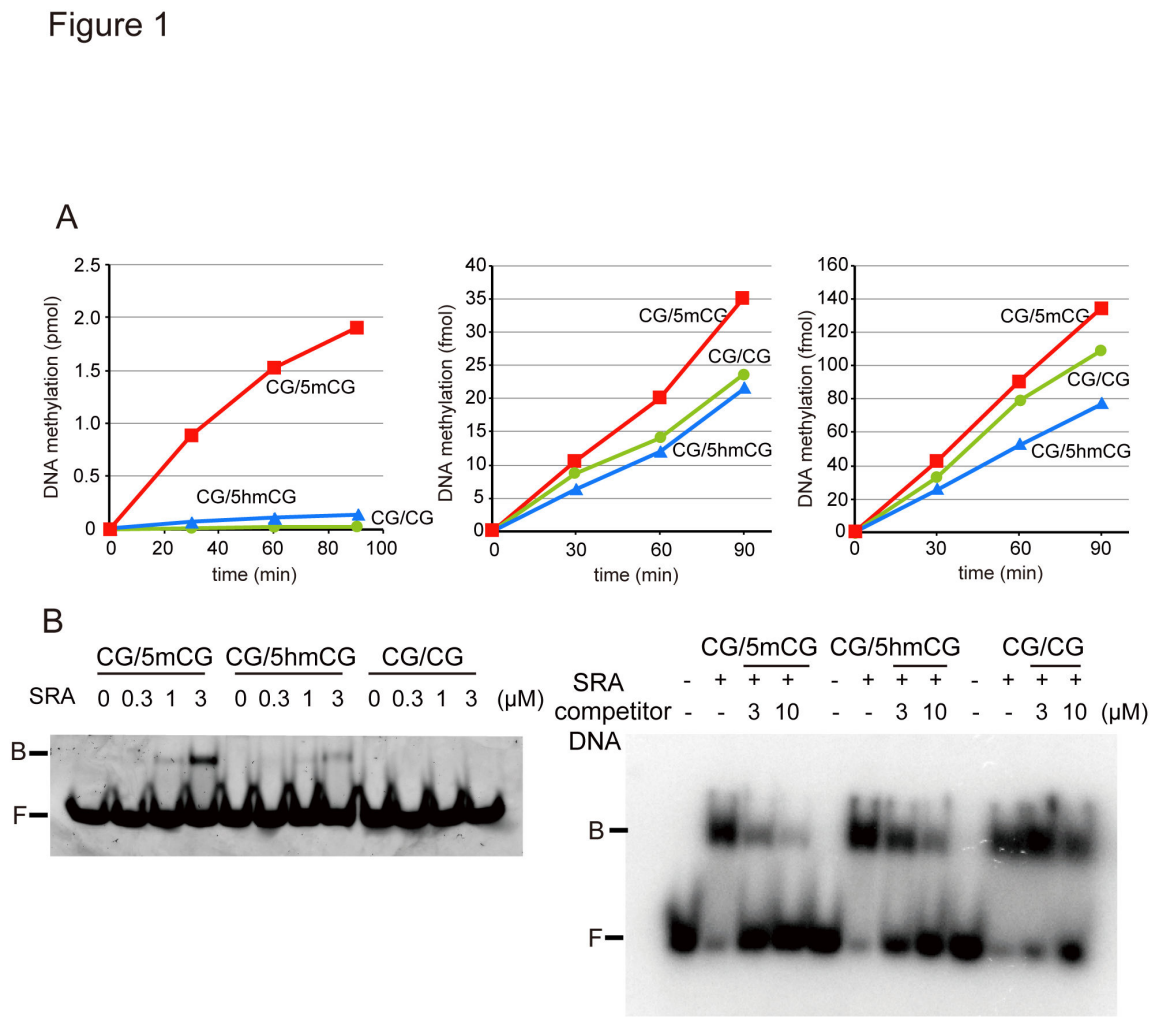
5hmC content is diluted during replication. **A**. Hemi-hydroxymethylated DNA (CG/5hmCG) is not a good substrate for Dnmt1. The DNA methylation activity of mouse Dnmt1, Dnmt3a, and Dnmt3b towards 35-bp unmethylated (CG/CG), hemi-methylated (CG/5mCG), or hemi-hydroxymethylated (CG/5hmCG) DNA was determined. **B**. Gel mobility shift assaying of the SRA domain of mouse Uhrf1. The indicated concentrations of SRA were incubated with either 12-bp CG/5mC, CG/5hmCG, or CG/CG, followed by electrophoresis (left panel). The complex of the SRA and ^32^P-labeled CG/5mCG was competed with the indicated amounts of non-labeled CG/5mCG, CG/5hmCG, or CG/CG DNA (right panel). DNA bound to SRA (B) and free DNA (F) are indicated.

Recent reports suggest that 5-hydroxymethylcytosine (5hmC) is an intermediate of demethylation. There are two possible models; one is active demethylation coupled with base-excision repair machinery [37], and the other is replication-dependent passive demethylation. According to the reports, hemi-hydroxymethylated DNA is not a good substrate for Dnmt1 [38,39]. As shown in Figure 1A, Dnmt1 scarcely methylated hemi-hydroxymethylated DNA (CG/5hmCG) compared to hemi-methylated DNA (CG/5mCG). The reaction rate for CG/5hmCG was calculated to be less than 1/10 of that for CG/5mCG from the slopes of the linear fitting curves. Two *de novo*-type DNA methyltransferases, Dnmt3a and Dnmt3b, showed almost identical DNA methylation activities towards un-methylated, hemi-methylated, and hemi-hydroxymethylated DNA (Figure 1A). 

In addition to Dnmt1, Uhrf1 is a prerequisite factor for the maintenance methylation during replication [8]. Recently, Frauer et al. reported that the SRA domain of Uhrf1 can specifically bind hemi-hydroxymethylated DNA with similar affinity to that for hemi-methylated DNA [40]. However, the pocket for binding 5mC is too narrow to accommodate 5hmC [9] (see Figure S2). In our case, though the SRA domain could bind to hemi-hydroxymethylated DNA, the affinity was lower than that for hemi-methylated DNA (Figure 1B). This was confirmed by the observation that an excess amount of hemi-hydroxymethylated DNA could not effectively compete with hemi-methylated DNA (Figure 1B, compare with the competition with hemi-methylated DNA). The SRA domain distinguishes hemi-5hmC DNA from hemi-5mC DNA. This finding is consistent with the report by Hashimoto et al. [39]. Assuming that flipping of 5mC and its binding to the binding pocket in the SRA domain of Uhrf1 are necessary steps for the maintenance methylation function, together with the observation that hemi-hydroxymethylated DNA is not a good substrate for Dnmt1, 5mC with the hydroxyl modification is not efficiently recognized as a substrate for the maintenance DNA methylation machinery during DNA replication. This may thus cause dilution of 5mC and 5hmC during DNA replication. Recently, it was reported that hydroxymethylcytosine is further oxidized to formylmethylcytosine and then to carboxymethylcytosine by Tet, and eventually demethylated through the base excision repair (BER) system [41]. However, the present study indicates that hydroxymethylcytosine can be passively demethylated during replication without further oxidization.

**Figure 2:**
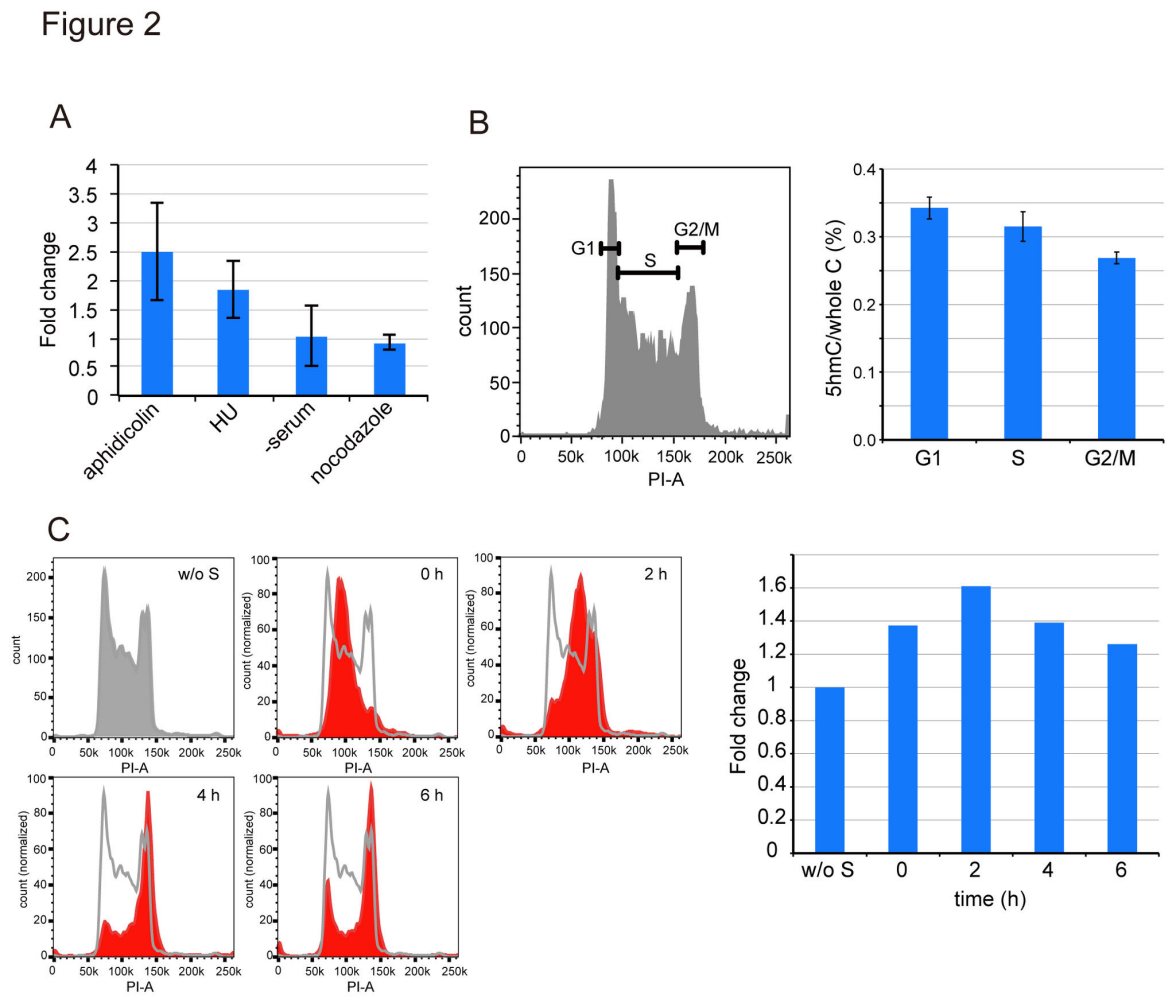
Cell cycle-dependent change in the 5hmC content. **A**. The 5hmC content in mESCs treated with aphidicolin, hydroxyurea, serum depletion, or nocodazole was determined by β-GT assaying. The values represent the fold change normalized as to that without treatment. The values for each treatment are averages ± SD (n=3). **B**. The 5hmC content in mESCs sorted by FACS (left panel) was determined (right panel). The values are averages ± SE (n=3). **C**. The non-synchronized (w/o S) and synchronized mESCs were collected after the indicated times and the 5hmC contents were determined. The left panels show the results of FACS analyses and the right panel the 5hmC content.

If the 5hmC removal is a replication-dependent event, the 5hmC content in mouse embryonic stem cells (mESCs) must be affected by cell cycle arrest due to the balance between the production and replication-dependent dilution of 5hmC. Therefore, the level of 5hmC in mESCs was determined in the presence of aphidicolin and hydroxyurea, which arrest cells at the S-phase, serum-free medium, which arrests cells at the G1/0-phase, and nocodazole, which arrests cells at the G2/M-phase, respectively (Figure 2A). Neither serum-free medium nor nocodazole affected the content of 5hmC as to that of proliferating mESCs. On the other hand, aphidicolin- or hydroxyurea-treated cells exhibited an about two-fold increase of the 5hmC level in the genome. 

To avoid the side effects of the inhibitors, the cells were sorted as to the G1-, S-, and G2/M-phases by FACS, and then the 5hmC contents were determined. The 5hmC contents were high at the G1- to S-phases and decreased at the G2/M-phase (Figure 2B). This is consistent with the results obtained in the effect of cell cycle inhibitor experiment. However, the 5hmC contents at the G1- and S-phases were about 1.4 and 1.2 fold higher, respectively, than that at the G2/M-phase, which was not as prominent as in Figure 2A. This can be due to that the FACS-sorted cells comprised a mixture of the broad range of the cells at the cell cycle stages, and thus the 5hmC content was averaged. To sort the cells more accurately, we next synchronized the cell stages by arresting the cells at the G1/S-phase with double thymidine block, released the cell proliferation, recovered the genome DNA after the indicated times, and then determined the 5hmC contents. As shown in Figure 2C, the level of 5hmC at time 0 (G1/S-phase) and after 2h culture (S-phase entered) had increased to 1.4 and 1.6 fold, respectively, compared to that without synchronization (w/o S). This finding supported the idea that 5hmC is diluted during replication.

### 5mC produced by *de novo*-type DNA methyltransferses Dnmt3a and Dnmt3b is the major substrate for hydroxymethylation in mESCs

5hmC is produced from 5mC by Tet enzymes [14,15]. Since 5hmC accumulated in the genome decreased during replication, as described above, the level of 5hmC in mESCs is expected to decrease as the cells proliferate. However, contrarily, the steady state content of 5hmC remains high [29]. This indicates that 5mC, which is a substrate for hydroxymethylation, is actively produced during one round of the cell cycle. We expected that not the 5mC sites maintained by Dnmt1 but 5mC newly produced by Dnmt3a and/or Dnmt3b is the target of hydroxylation. 

**Figure 3:**
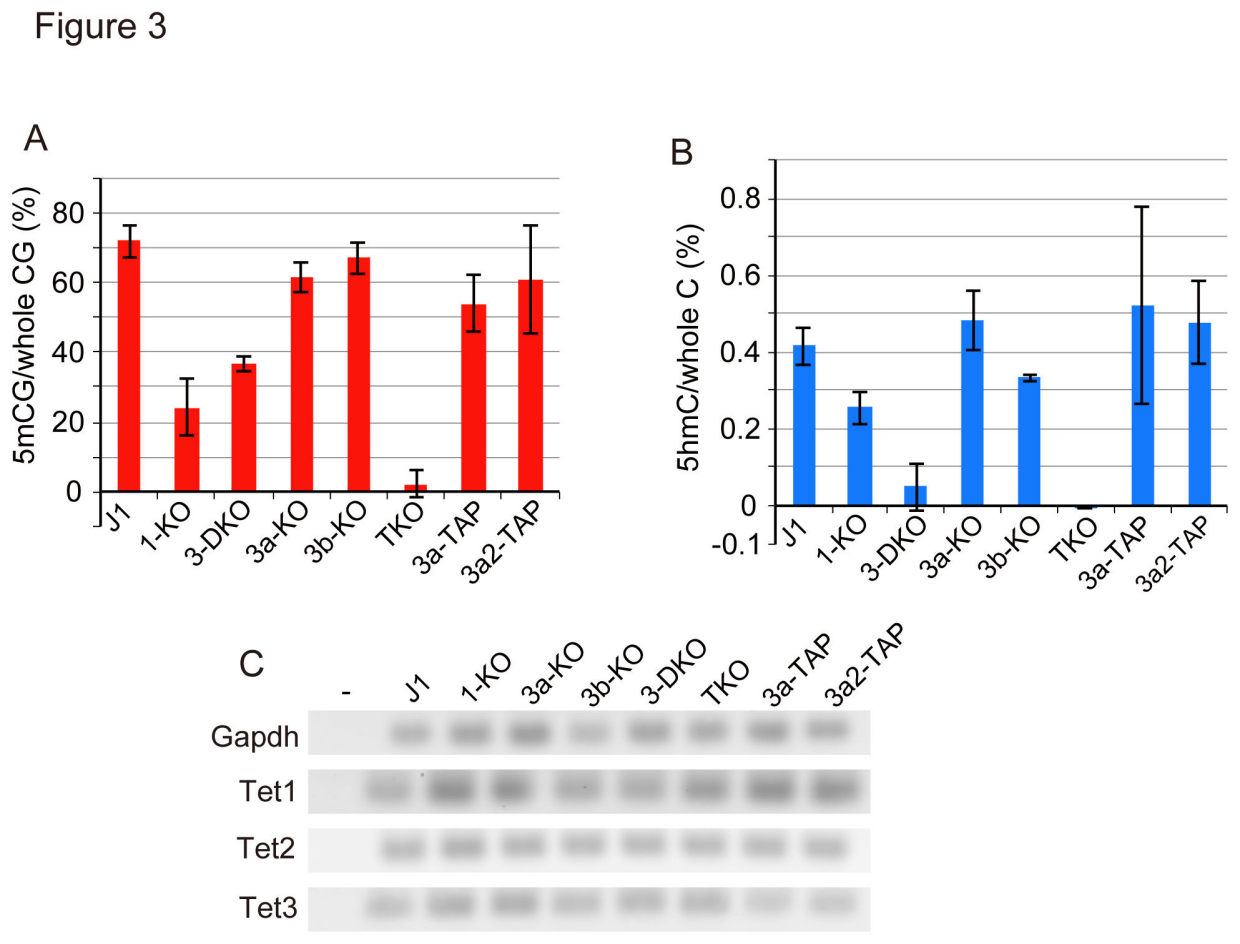
Dnmt3a and Dnmt3b mainly provide 5mC for the hydroxymethylation in mESCs. The 5mC, 5hmC, and Tet mRNA contents of J1 parent, *Dnmt1* (1-KO), *Dnmt3a* and *Dnmt3b* (3-DKO), *Dnmt3a* (3a-KO), *Dnmt3b* (3b-KO), and *Dnmt1*, *Dnmt3a* and *Dnmt3b* (TKO) knockout mESCs, and ectopically expressed TAP-tagged Dnmt3a (3a-TAP) or Dnmt3a2 (3a2-TAP) in 3-DKO mESCs were determined. **A**. The 5mC contents (%) were determined as M.SssI methylation ability from a standard curve (Figure S1A). **B**. The 5hmC contents were determined from the standard curve obtained on β-GT assaying (Figure S1B). The values are the averages ± SD determined for three independent genomic DNA samples. **C**. Relative mRNA expression of Tet1, Tet2, and Tet3 was evaluated by semi-quantitative RT-PCR. (-) indicates the product of PCR without the template.

To determine which DNA methyltransferase is responsible for producing 5mC as the substrate for Tet to generate 5hmC in mESCs, we determined the levels of 5mC and 5hmC in *Dnmt1*, *Dnmt3a*, or/and *Dnmt3b*-knockout mESCs (Figure 3A and B). Since triple-knockout mESCs (TKO) lack substrate 5mC [25], 5hmC was below the detection level in mESCs [29] (see Figure 3B). Although the *Dnmt1*-knockout mESCs (1-KO) were impaired in the maintenance methylation, and thus the DNA methylation level was significantly decreased after 10 passages, the reduction in the level of 5hmC was not so prominent compared to that of 5mC. Knockout of either *Dnmt3a* (3a-KO) or *Dnmt3b* (3b-KO) did not affect either the 5mC or 5hmC level compared to those in the parental mESCs. Surprisingly, however, knockout of both *Dnmt3a* and *Dnmt3b* (3-DKO) [1] significantly decreased the 5hmC level to almost below the detection level. In such cells, about half the 5mC level in the parent mESCs remained, which was the result of maintenance methylation by Dnmt1 (Figure 3A and B). The levels of transcripts produced from the *Tet1*, *Tet2*, and *Tet3* genes were not significantly changed compared to those in *Dnmt1*, *Dnmt3a*, or/and *Dnmt3b* knockout mESCs (Figure 3C). Ectopic expression of Dnmt3a or Dnmt3a2, a short form of Dnmt3a and expresses dominantly in mESCs [4], with a TAP-tag added to their C-termini, restored the 5hmC level in 3-DKO mESCs (Figure 3B). These results clearly indicate that *de novo*-produced 5mC is a selective substrate for hydroxylation by Tet in mESCs.

### 5: hmC-enriched regions in mESs

**Figure 4:**
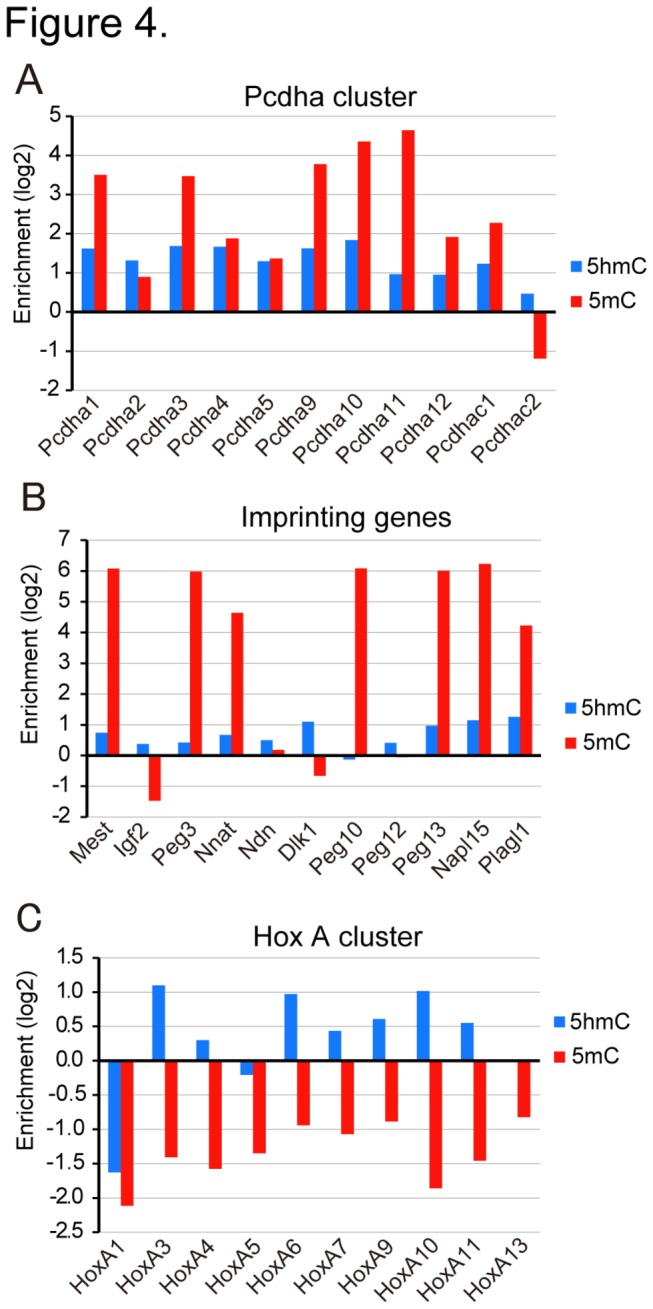
Enrichment of 5mC and 5hmC in specific promoters. 5hmC (blue bars) and 5mC (red bars) were determined by DNA microarray analysis in the promoters of the *Pcdha* gene cluster (**A**), maternal imprinting genes (**B**), and *HoxA* gene cluster (**C**). The abscissas indicate enrichment of 5hmC or 5mC on a log_2_ scale.

Analyses of 5hmC and 5mC in 3-DKO cells demonstrated that the 5mC produced by Dnmt3a or Dnmt3b is selectively 5-hydroxylated in mESCs. Recent genome wide analysis of 5hmC demonstrated that 5hmC is enriched at the transcription start sites and gene bodies in mESCs [17,18]. To determine the regions of hydroxymethylation, we performed microarray analysis to identify the regions enriched with 5hmC. Both 5hmC- and 5mC-containing DNA fragments were selectively precipitated by the chemical labeling method [31] and with the recombinant methylated DNA-binding domain of MBD1 [32], respectively, and then were hybridized with mouse CpG island arrays. A list of the genes containing 5hmC and 5mC, with annotations, is presented as Table S3. Gene ontology analysis demonstrated that most of the genes containing 5hmC were related to the developmental process (Figure S3), which is consistent with previous reports [17,18]. Consistent with other genome wide analyses, we found that *Pcdha* and *Hoxa* gene clusters are enriched with 5hmC [17,42]. We also found that the promoters of *Pcdha* genes and some maternally imprinted genes (*Mest, Peg3, Nnat, Ndn, Peg13, Napl15*, and *Plagl1*) are enriched with both 5hmC and 5mC. The promoters of *Igf2* and *Dlk1* were poor in 5mC and rich in 5hmC (Figure 4A-C). The promoters of *HoxA* genes are reported to be enriched with histone H3 tri-methylated at K27 (H3K27me3) and poor in 5mC [43,44]. As 5hmC is generated from 5mC as a substrate, it is reasonable to speculate that the 5mC in *HoxA* cluster regions is susceptible to Tet catalysis, and thus hydroxylated as soon as the regions are methylated. 

**Figure 5:**
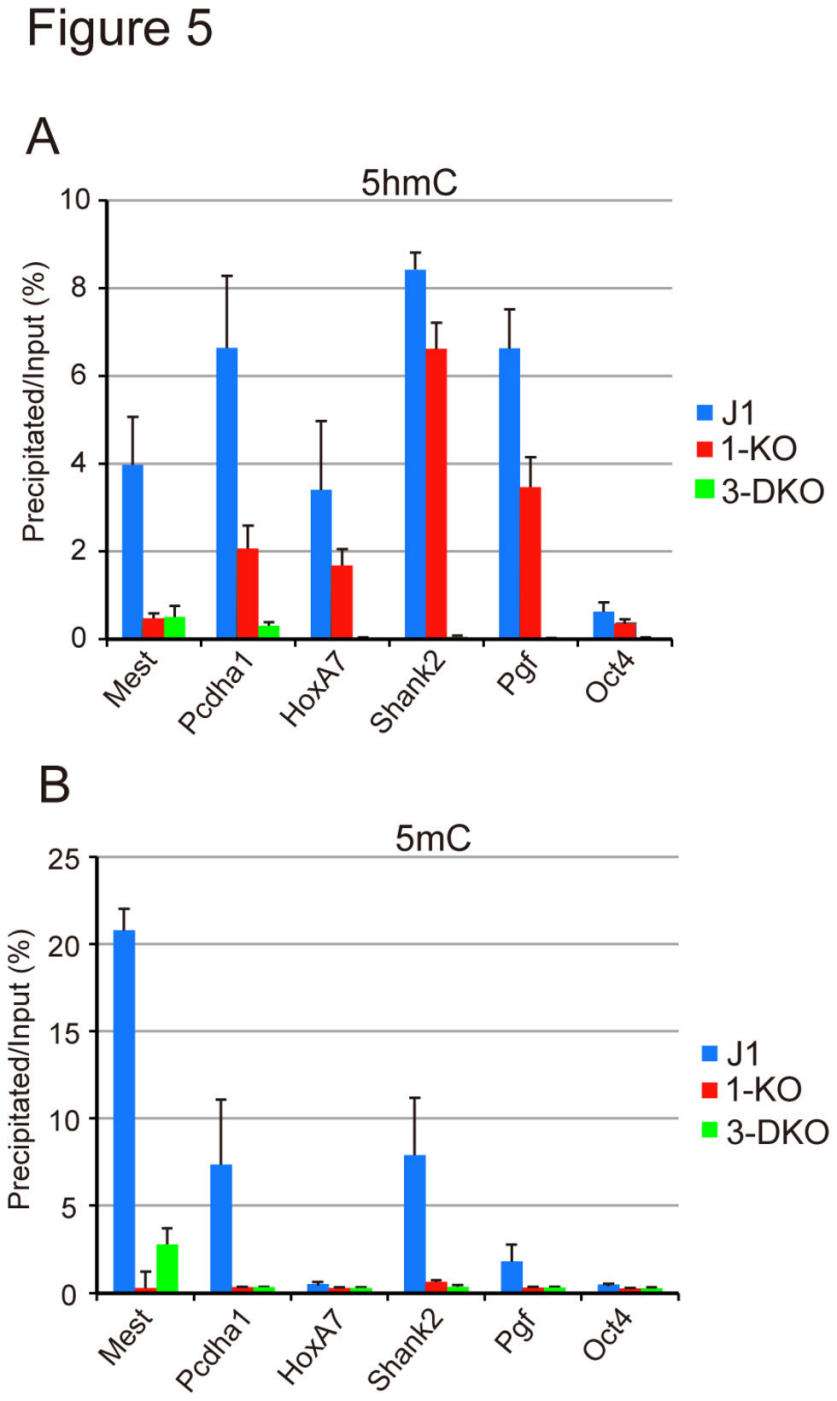
Dnmt3a and Dnmt3b-dependent 5mC are responsible for the production of 5hmC. The 5hmC (**A**) and 5mC (**B**) contents of J1 (blue bars), *Dnmt1* (1-KO, red bars), and *Dnmt3a* and *Dnmt3b* (3-DKO, light green bars) knockout mESCs were determined by q-PCR in the promoters of five representative 5hmC-enriched genes. The values are the averages + SD determined for three independent genomic DNA samples.

The 5hmC-positive promoters of five genes, i.e. *Mest*, *Pcdha1*, *HoxA7*, *Shank2*, and *Pgf*, which are reported to have high 5hmC contents [17,42], were chosen and quantitated as to 5hmC and 5mC enrichment by qPCR. The depletion of not Dnmt1 but both Dnmt3a and Dnmt3b selectively reduced 5hmC in all the promoters of the genes examined in mESCs except for *Mest* (Figure 5A). In *Mest*, not only double-knockout of *Dnmt3a* and *Dnmt3b* (3-DKO), but also *Dnmt1* knockout (1-KO) reduced the 5hmC level. Despite the exception of *Mest*, the results support the idea that the sites of *de novo* DNA methylation by Dnmt3a and Dnmt3b are the major target of hydroxylation, and that the methylated sites maintained by Dnmt1 limitedly contribute to the production of 5hmC. As both *Dnmt1* knockout (1-KO), and *Dnmt3a* and *Dnmt3b* knockout (3-DKO) mESCs exhibited a drastically decreased 5mC level in these examined regions, these sites are susceptible to maintenance and *de novo* methylation (Figure 5B). However, not *Dnmt1* knockout, but only *Dnmt3a* and *Dnmt3b* knockout significantly reduced the 5hmC level (Figure 5A). The results further support that Dnmt3a and Dnmt3b-methylated CpGs are the major target for hydroxylation in mESCs.

### Dnmt3a and Dnmt3b are localized in 5hmC-enriched regions

**Figure 6:**
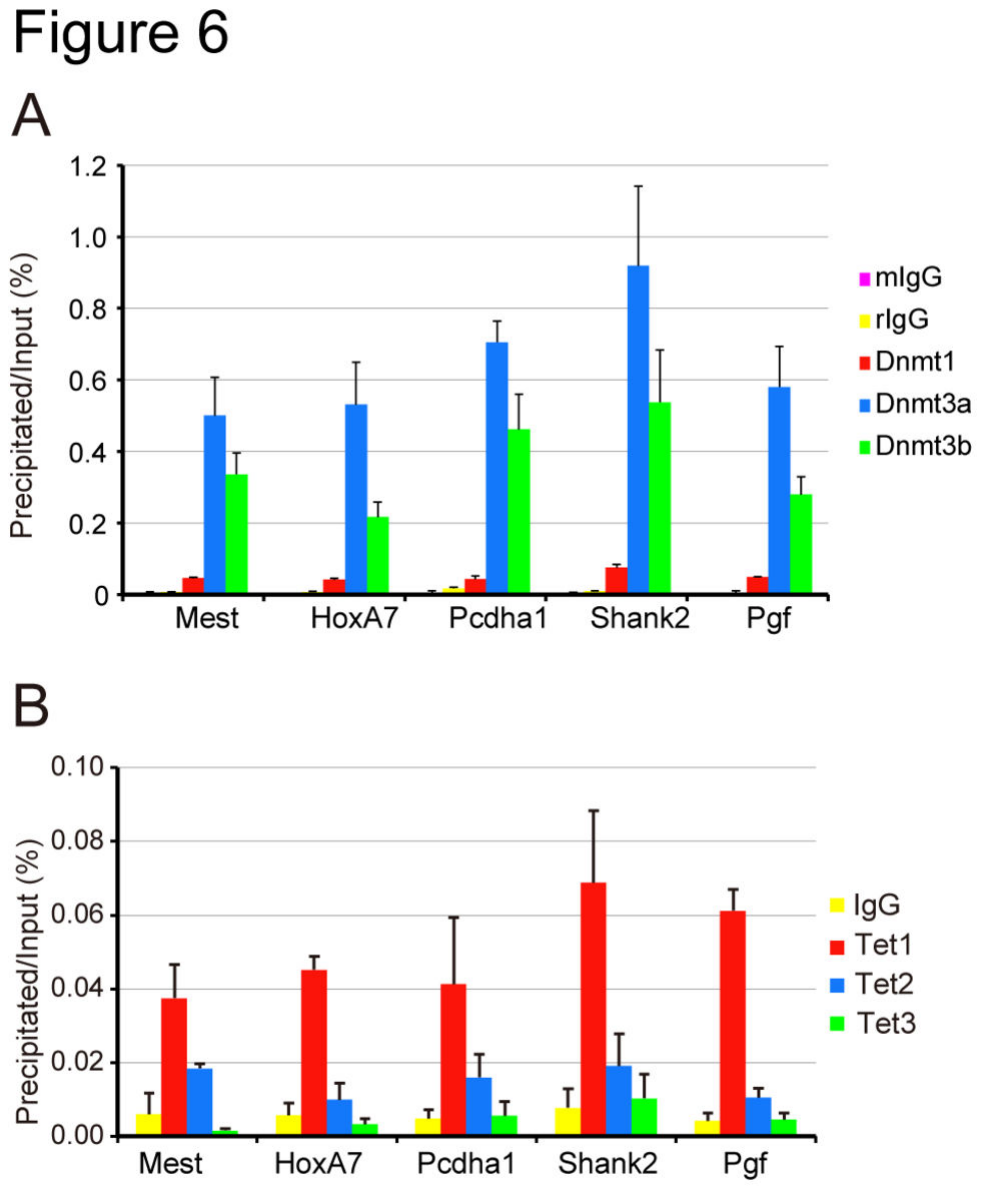
Dnmt1, Dnmt3a, Dnmt3b, and Tet1 are recruited to 5hmC-enriched promoters. The occupancy of Dnmt1, Dnmt3a, and Dnmt3b (**A**), and Tet1, Tet2, and Tet3 (**B**) was determined by ChIP-qPCR in the promoters of the 5hmC-enriched genes shown in Figure 4. The values are the averages + SD determined for three independent DNA samples.

In the present study, we have shown that Tet in mESCs selectively hydroxylates the 5mC produced by Dnmt3a and Dnmt3b. We next examined whether or not Dnmt3a/Dnmt3a2 and Dnmt3b are localized in 5hmC-enriched regions in mESCs. To this end, we performed ChIP-qPCR analyses to quantitate the enrichment of Dnmt1, Dnmt3a/3a2, and Dnmt3b in the promoters of the five 5hmC-positive genes shown in Figure 5. As expected, both Dnmt3a/Dnmt3a2 and Dnmt3b were localized in all the examined regions where 5hmC was enriched (Figure 6A), while Dnmt1 was not significantly localized to the promoters of the five selected genes. On the other hand, not Tet2 and Tet3, but only Tet1 was positively accumulated in the regions examined for 5hmC (Figure 6B). This could be a reflection of the different expression levels and target genes of Tet1, Tet2, and Tet3 in mESCs [29].

## Discussion

### Dnmt1- and Uhrf1-dependent passive demethylation

In the present study we have confirmed that maintenance-type DNA methyltransferase Dnmt1 scarcely methylates hemi-hydroxymethylated DNA using highly purified Dnmt1 [28] (Figure 1A). In addition, we have shown that hemi-methylated DNA binding domain SRA of Uhrf1, which is a prerequisite factor for maintenance DNA methylation in mESCs [8], less effectively recognized hemi-hydroxymethylated DNA than hemi-methylated DNA (Figure 1B). The present results are consistent with those reported by Hashimoto et al. [39]. However, this observation is contrary that of Frauer et al. [40], who reported that the SRA domain of Uhrf1 selectively binds hemi-hydroxymethylated DNA as well as hemi-methylated DNA. Recently, Uhrf1 was reported to be the reader for 5hmC in mESCs [45]. The reason for this discrepancy is not clear, however, our present finding does not eliminate that Uhrf1 is the reader of 5hmC but indicates that the affinity of Uhrf1 (SRA) towards 5hmC is low. The difference in the sequences and/or the lengths of the DNA used may partly be the reason for the discrepancy. 

Due mainly to the substrate recognition of Dnmt1 and possibly by the binding selectivity of Uhrf1, the 5hmC position cannot be methylated in the daughter strand after replication, and thus the replicated DNA is demethylated. Recently, replication-dependent depletion of 5hmC in mouse primordial germ cells [46] and in the male pronuclei of fertilized eggs [47] was reported. Our present findings that Dnmt1 cannot methylate hemi-hydroxymethylated DNA, and that Uhrf1 cannot bind 5hmC provides the molecular basis of this genome-wide passive demethylation.

### *De novo* methylated sites are selectively hydroxylated in mESCs

We have shown that the major substrate, 5mC, for hydroxylation is supplied through *de novo* DNA methylation by Dnmt3a (Dnmt3a2) and Dnmt3b in mESCs (Figure 3). Since 5hmC seems to be diluted to half during replication, the reduced level of 5hmC must be supplied in a single round of the cell cycle, i.e. after replication to the next replication. It is reasonable that the expression of high levels of Dnmt3a2 and Dnmt3b, compared to in ordinary somatic cells, in mESCs [4,5] supplies 5mC for hydroxylation. These observations indicate that the removal and generation of 5hmC are cell cycle-dependent, and this idea is illustrated in Figure S4.

*Dnmt3a* and *Dnmt3b* are reported to be necessary for embryo development and terminal differentiation of mESCs [1,48], which may yield the methylation state of the genome for proper terminal differentiation. Recombinant Dnmt3a and Dnmt3b, on the other hand, preferably methylate the linker portion of nucleosomes when that region is naked and exposed [49,50]. Dnmt3a2 and Dnmt3b in mESCs may methylate rather naked or euchromatic regions of the genome, most of which are undesirable as to maintenance of pluripotency and/or terminal differentiation, during the cell cycle. The hydroxymethylation by Tet could be a protection tool for preventing aberrant methylation of the genome in mESCs. 

Interestingly, many of the *HoxA* genes in the *HoxA* gene cluster were found to be highly hydroxymethylated, whilst the region was quite poor in 5mC (Figure 4C). It is well known that their expression is not regulated by DNA methylation but positively and negatively regulated by *TrxG* and *PcG* through K4 and K27 trimethylation of histone H3, respectively [51]. Although a negligible amount of 5mC was found in the *HoxA* gene cluster and individual *HoxA7* genes, the 5hmC level was significantly high (see Figures 4C and 5). This suggests that the sites of aberrant methylation by Dnmt3a2 or/and Dnmt3b are hydroxylated in mESCs to keep the sites hypomethylated. Actually, in somatic fibloblasts and monocytes, the *HoxA* gene cluster is heavily methylated and silent [43]. *Pcdh* genes are highly expressed in neurons and determines the properties of neurons, and their expression is regulated by DNA methylation [52]. Since there is a high level of 5hmC in the brain [29], it is reasonable that the promoters of *Pcdha* genes are rich in both 5mC and 5hmC. The methylation and hydroxymethylation in *Pcdha* genes must be dynamically regulated in mESCs and for terminal differentiation. Neurons, however, are post-mitotic, and thus instead of passive demethylation via replication, the base excision repair mechanism may be used for demethylation [15,41]. The genes related to development and differentiation are enriched in 5hmC, as found on gene ontology analysis (Figure S3), which supports that Tet enzymes protect such genes from DNA methylation to maintain the pluripotency of mESCs.

Dnmt3a2 and Dnmt3b were significantly localized in 5hmC-enriched regions. On the other hand, however, only Tet1, which is the major Tet expressed in mESCs [27], was positively enriched in the examined regions, however, its amount was not prominent. Recent genome-wide analysis showed that Tet seems to be absent from 5hmC-enriched regions [13,37]. One possible explanation is that Tet leaves its target soon after converting 5mC to 5hmC to prevent further oxidation. 

## Supporting Information

Figure S1 **Calibration curves for the determination of 5mC and 5hmC.** **A**. M.SssI methylation activity towards 200 ng of standard DNA mixed with 0:1, 1:4, 2:3 and 4:1 of um-methylated and full-methylated DNA. **B**. Glucosyltransferase activity of β-GT towards 200 ng of un-hydroxylated DNA with 0, 0.1, 0.4, and 1 ng of fully-hydroxylated DNA.
(PDF)

Figure S2 **The binding pocket for 5mC of the SRA domain of Uhrf1 cannot accommodate hemi-5hmC.** The figure demonstrates the tight recognition of 5mC by the crystal structure of the SRA domain of Uhrf1 in a complex with CG/5mCG (PDB code; 2ZKD). The flipped 5mC base and the protein side chains that are critical for 5mC recognition are shown as stick models in purple and green, respectively. The yellow dotted lines represent van der Waals contacts (3.5 - 4.1 Å) with the methyl group of 5mC.
(PDF)

Figure S3 **Gene ontology analysis of 5hmC- and 5mC-enriched genes.** The 5hmC- (**A**) and 5mC- (**B**) enriched genes were analyzed using DAVID functional annotation tools (Huang, D. W., Sherman, B. T., & Lempicki, R. A. Systematic and integrative analysis of large gene lists using DAVID Bioinformatics Resources. *Nature Protoc*. **4**, 44-57, 2009). The X-axes indicate p-values.
(PDF)

Figure S4 **Cell cycle-dependent hydroxylation in mESc.** DNA-methylated sites created by Dnmt3a and Dnmt3b during proliferation were actively hydroxylated and were diluted during replication, as hemi-hydroxymethylated DNA is not a good substrate of the maintenance methylation machinery, Dnmt1 and the SRA of Uhrf1.
(PDF)

Table S1 **Primer sets for the semi-quantitative PCR and the amplification conditions.**
(DOCX)

Table S2 **Primer sets for qPCR.**
(DOCX)

Table S3 **List of the genes precipitated by click chemistry (5hmC) and MBD1 (5mC).** 5hmC-containing DNA fragments were precipitated without amplification (5hmC Direct) or after amplification (5hmC IVT).
(XLSX)
