# Supplementary figures and images for: Cell Cycle-Dependent Turnover of 5-Hydroxymethyl Cytosine in Mouse Embryonic Stem Cells
Source: PLoS One. 2013 Dec 10;8(12):e82961. doi: 10.1371/journal.pone.0082961 (PMC3858372; doi:10.1371/journal.pone.0082961)

Supplementary Figure S1.

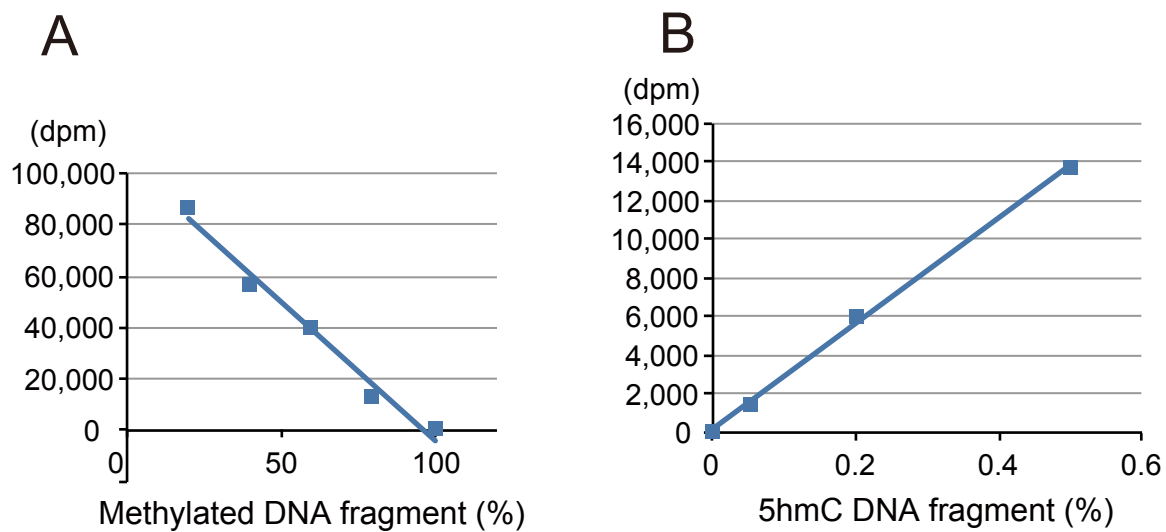

Supplement: Figure S1 — Calibration curves for the determination of 5mC and 5hmC. A. M.SssI methylation activity towards 200 ng of standard DNA mixed with 0:1, 1:4, 2:3 and 4:1 of um-methylated and full-methylated DNA. B. Glucosyltransferase activity of β-GT towards 200 ng of un-hydroxylated DNA with 0, 0.1, 0.4, and 1 ng of fully-hydroxylated DNA. (PDF) [file pone.0082961.s001.pdf]

Supplementary Figure S2.

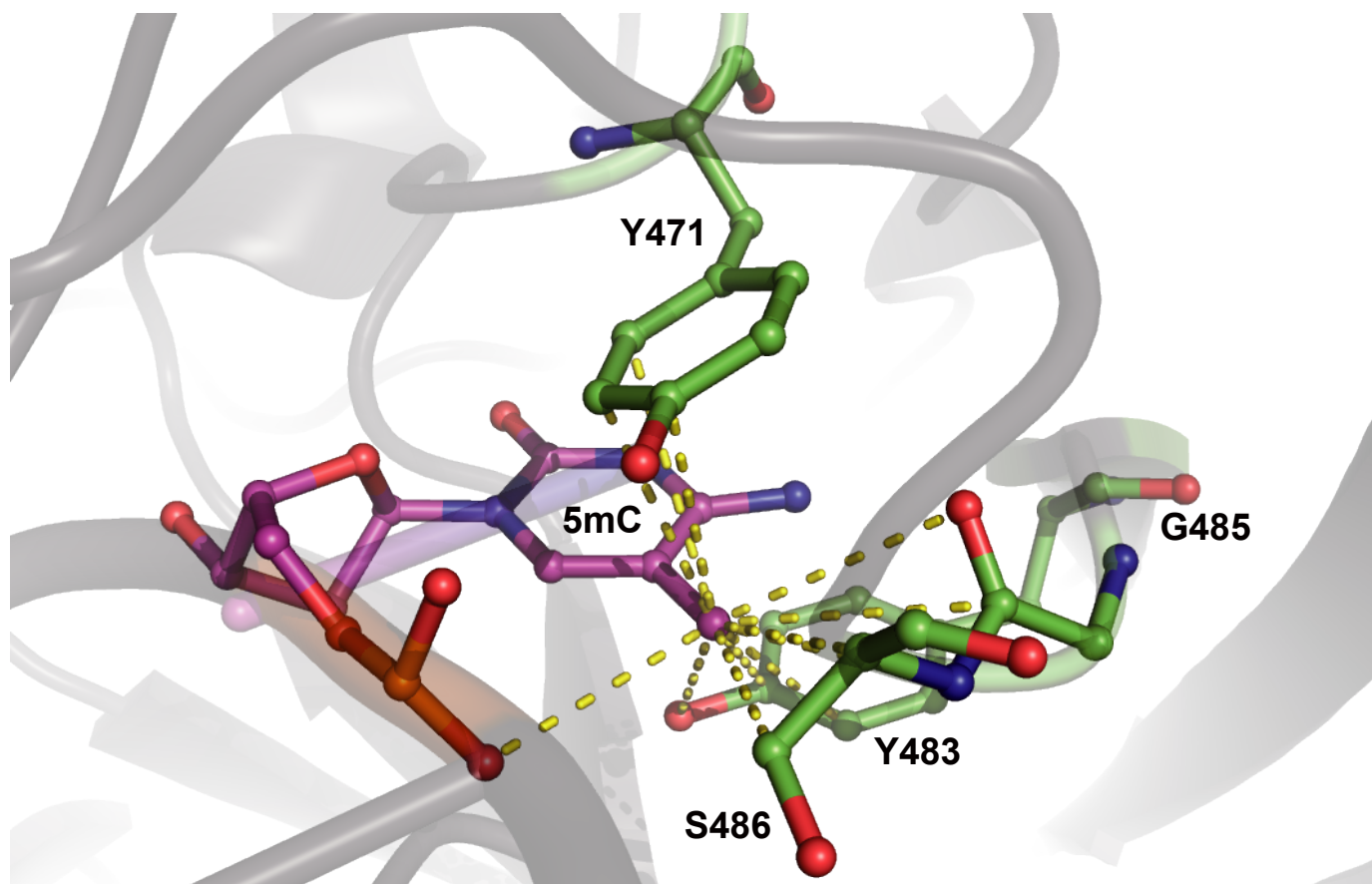

Supplement: Figure S2 — The binding pocket for 5mC of the SRA domain of Uhrf1 cannot accommodate hemi-5hmC. The figure demonstrates the tight recognition of 5mC by the crystal structure of the SRA domain of Uhrf1 in a complex with CG/5mCG (PDB code; 2ZKD). The flipped 5mC base and the protein side chains that are critical for 5mC recognition are shown as stick models in purple and green, respectively. The yellow dotted lines represent van der Waals contacts (3.5 - 4.1 Å) with the methyl group of 5mC. (PDF) [file pone.0082961.s002.pdf]

Supplementary Figure S3.

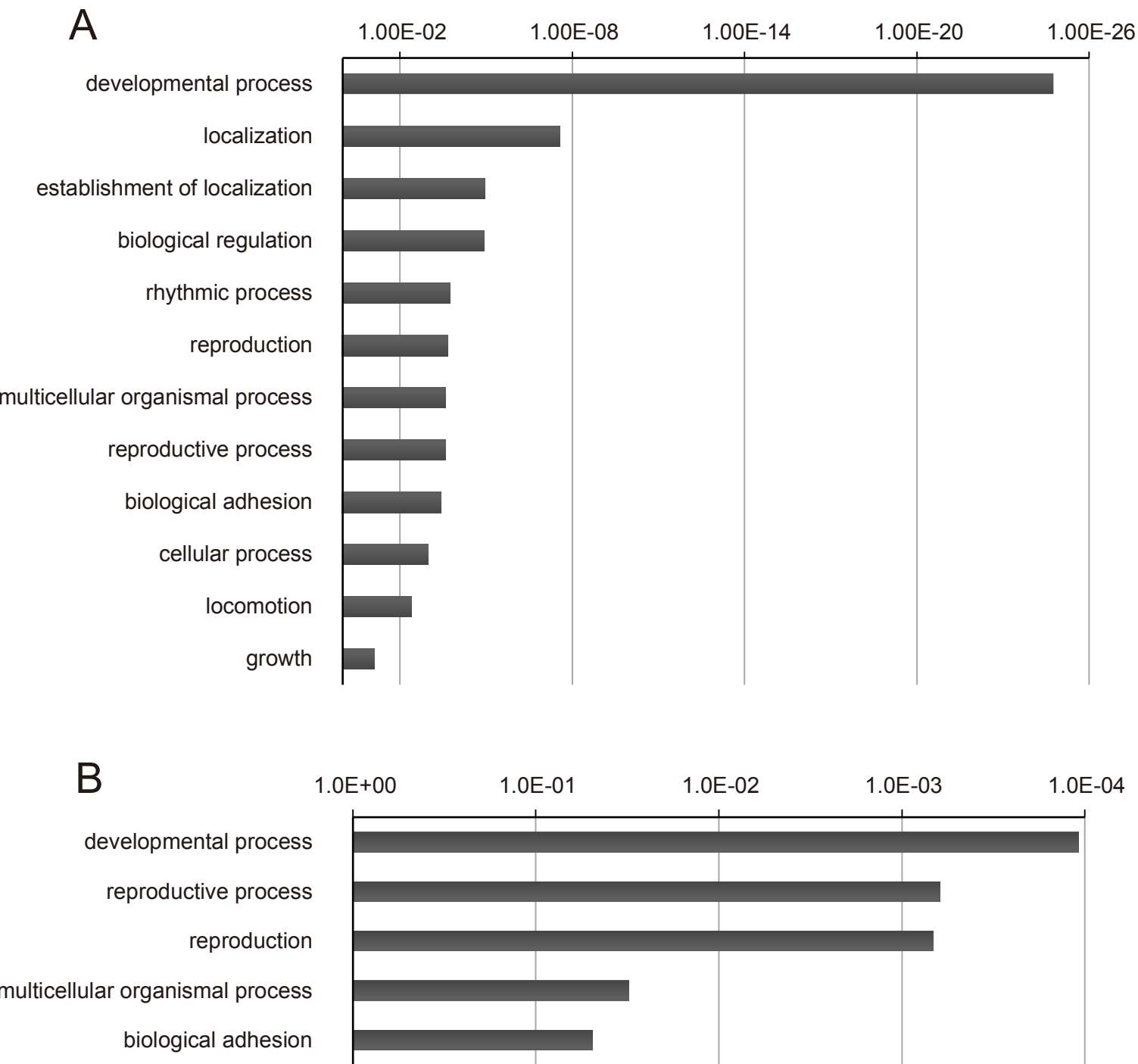

Supplement: Figure S3 — Gene ontology analysis of 5hmC- and 5mC-enriched genes. The 5hmC- (A) and 5mC- (B) enriched genes were analyzed using DAVID functional annotation tools (Huang, D. W., Sherman, B. T., & Lempicki, R. A. Systematic and integrative analysis of large gene lists using DAVID Bioinformatics Resources. Nature Protoc. 4, 44-57, 2009). The X-axes indicate p-values. (PDF) [file pone.0082961.s003.pdf]

Supplementary Figure S4

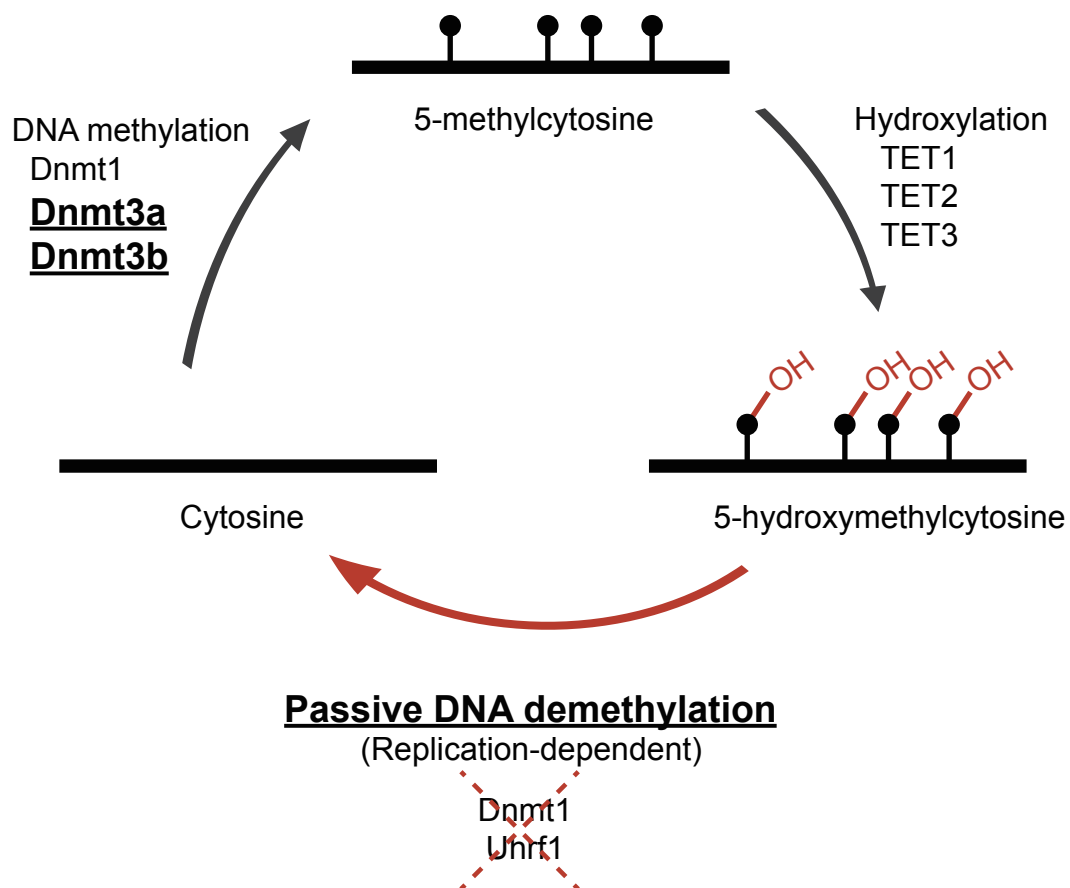

Supplement: Figure S4 — Cell cycle-dependent hydroxylation in mESc. DNA-methylated sites created by Dnmt3a and Dnmt3b during proliferation were actively hydroxylated and were diluted during replication, as hemi-hydroxymethylated DNA is not a good substrate of the maintenance methylation machinery, Dnmt1 and the SRA of Uhrf1. (PDF) [file pone.0082961.s004.pdf]
