# Supplementary material for: Cell Cycle-Dependent Turnover of 5-Hydroxymethyl Cytosine in Mouse Embryonic Stem Cells
Source: PLoS One. 2013 Dec 10;8(12):e82961. doi: 10.1371/journal.pone.0082961 (PMC3858372; doi:10.1371/journal.pone.0082961)
Supplement: Table S1 — Primer sets for the semi-quantitative PCR and the amplification conditions. (DOCX) [file pone.0082961.s005.docx]

Table S1. Primer sets for the semi-quantitative PCR and the amplification conditions.

Gene Primer sequences Temperature Cycles

Conditions

*Tet1* fw GAGCCTGTTCCTCGATGTGG 95ºC 10 sec

rv TTAGTGTTGTGTGAACCTGATTTATTGT 69ºC 10 sec 25

72ºC 20 sec

*Tet2* fw AACCTGGCTACTGTCATTGCTCCA 95ºC 10 sec 30

rv ATGTTCTGCTGGTCTCTGTGGGAA 72ºC 20 sec

*Tet3* fw TCCGGATTGAGAAGGTCATC 95ºC 10 sec

rv CCAGGCCAGGATCAAGATAA 60ºC 10 sec 30

72ºC 2 min

*Gapgh* fw GTGTTCCTACCCCCAATGTGT 95ºC 10 sec

rv ATTGTCATACCAGGAAATGAGCTT 60ºC 10 sec 20

72ºC 20 sec

Prior to the temperature cycles, all the samples were preheated at 95ºC for 2 min.

“fw” and “rv” indicate forward and reverse primers, respectively.
