# Supplementary material for: Cell Cycle-Dependent Turnover of 5-Hydroxymethyl Cytosine in Mouse Embryonic Stem Cells
Source: PLoS One. 2013 Dec 10;8(12):e82961. doi: 10.1371/journal.pone.0082961 (PMC3858372; doi:10.1371/journal.pone.0082961)
Supplement: Table S2 — Primer sets for qPCR. (DOCX) [file pone.0082961.s006.docx]

Table S2. Primer sets for qPCR.

Gene Primer sequences

*HoxA7*^1^ fw GAGAGGTGGGCAAAGAGTGG

rv CCGACAACCTCATACCTATTCCTG

*Mest* fw TATCATGGGCTAAGGGCTTG

rv CGGCACCCACTTCTTTTCTA

*Oct4*^1^ fw GGCTCTCCAGAGGATGGCTGAG

rv TCGGATGCCCCATCGCA

*Pcdha1* fw CGCTGATGAAGGTGTTAATGGT

rv ATCACCGATCACTCTAATTTCTCCT

*Pgf* fw CTAGTCGGCCGCTGGTTTT

rv ACTCAGGTCTCACCGCCAAC

*Shank2* fw GATGCCCTGCTTGCTCTCTC

rv AGCCCACCCCATATTCTGTCT

^1^Sequences were taken from reference 35 in the text.

“fw” and “rv” indicate forward and reverse primers, respectively.
